# Antidiabetic and Hypolipidaemic Action of Finger Millet (*Eleusine coracana*)*-*Enriched Probiotic Fermented Milk: An *in vivo* Rat Study

**DOI:** 10.17113/ftb.58.02.20.6308

**Published:** 2020-06

**Authors:** Jinal Kesharbhai Chaudhary, Sreeja Mudgal

**Affiliations:** Dairy Microbiology Department, SMC College of Dairy Science, Anand Agricultural University, Anand 388 110, Gujarat, India

**Keywords:** finger millet, antidiabetic potential, functional food, probiotic, fermented milk

## Abstract

**Research background:**

Diabetes is a chronic multi-system disease having long term consequences to the health of people suffering from it. This study investigates the role of finger millet (*Eleusine coracana*)-enriched probiotic fermented milk in alleviating the diabetic complications in streptozotocin-induced diabetic rats.

**Experimental approach:**

The probiotic strain used in the study was *Lactobacillus helveticus* MTCC 5463. Study comprised six groups each containing 6 Sprague Dawley rats: two controls (nondiabetic and diabetic), and four diabetic groups fed finger millet-enriched probiotic fermented milk, probiotic fermented milk, finger millet flour or metformin (standard drug). Samples were administered orally for four weeks, and parameters associated with diabetic disorders were studied.

**Results and conclusions:**

Oral administration of finger millet-enriched milk significantly (p<0.001) decreased (64.26%) the fasting blood glucose level of diabetic rats compared to metformin (56.43%) and diabetic groups receiving the probiotic fermented milk (18.88%) and finger millet flour (47.14%) after four weeks of treatment. The finger millet-enriched milk significantly ameliorated the diabetic symptoms polyphagia and polydipsia and improved body mass. In diabetic control group, body mass was reduced up to 15.60% at the end of experiment, while in the group receiving the probiotic fermented milk, body mass significantly (p<0.0001) increased by about 5-30%. Significant (p<0.0001) reduction in total cholesterol, triglyceride levels in the groups treated with finger millet flour, finger millet-enriched probiotic fermented milk and probiotic fermented milk was observed compared to diabetic control rats. The probiotic fermented milk enriched with finger millet caused significant (p<0.05) decrease in low-density lipoprotein and very-low-density lipoprotein levels (p<0.0001) and insignificant increase in high-density lipoprotein level. A reversal of altered activities of hepatic marker enzymes aspartate transaminase and alanine transaminase was observed in the group receiving the probiotic milk enriched with finger millet. Histological observations of pancreatic tissues and liver showed that the enriched milk prevents more severe changes in the acinar cells and ameliorated the inflammation and alteration in the liver structure to some extent. Therefore, the finger millet-enriched probiotic fermented milk can be a potential functional food in the management of diabetes.

**Novelty and scientific contribution:**

This is the only paper reporting about the antidiabetic potential of finger millet-enriched milk fermented using probiotic *Lactobacillus helveticus* MTCC 5463 and *Streptococcus thermophilus* MTCC 5460. It also shows the synergistic antidiabetic effect of milk and finger millet used in combination, thus offering a novel functional food.

## INTRODUCTION

Diabetes mellitus is a metabolic disorder characterized by hyperglycaemia, impaired lipid and lipoprotein metabolism, oxidative stress and sub-clinical inflammation, which affect the quality of life of patients including social, psychological well-being as well as financial aspects (*1*). According to International Diabetes Federation estimates, the number of people suffering from diabetes mellitus is expected to rise to 642 million by 2040. A great change in dietary habits leading to overweight and obesity, together with physical latency is said to be the major reasons for substantial increase in the worldwide diabetes burden (*1*). Consumption of healthy foods and beverages and physical activity can prevent the occurrence of diabetes mellitus, particularly type 2 (T2DM). Mounting clinical evidence demonstrates that T2DM and the associated complications can be prevented or delayed in high risk people through regular intake of functional foods that can have an impact on glycaemic management through blood pressure regulation, activation of antioxidant enzymes, effect on gut microbiota, and suppress overproduction of pro-inflammatory cytokines during diabetes (*2*). Moreover, the use of functional foods as a supportive medical aid for prevention and management of diseases has steadily increased over the last few decades as a means of advancing health and emotional well-being, and has been increasingly practiced in cases wherever patients obtained relief of symptoms related to chronic illness and aftermath of conventional medication. Hence, in the process of development of a functional food, it is important to identify the health effects of foods through studies and communicate them to the consumers.

Probiotics are reported to play a vital role in the prevention of diabetes mellitus. A number of short-term randomized controlled trials have demonstrated the effects of probiotics and prebiotics on insulin sensitivity, inflammatory markers and glucose tolerance (*3*, *4*). Antidiabetic effect of probiotic strains of *Lactobacillus* and *Bifidobacterium* has been examined in animal and human studies (*5*). The results of some of the studies revealed that probiotic treatment can reduce blood glucose levels in diabetic patients (*6*). Intake of probiotic bacteria through dairy foods is considered the most appropriate way to re-establish the intestinal microflora balance. Among the various dairy food products, the fermented ones have been the most preferred delivery vehicle for probiotics. Even though fermented milk is considered superior in its nutritional and health benefits, it is deficient in micronutrients such as iron and vitamin C and dietary fibre known for their prebiotic effect. Such deficiency can be overcome through food fortification, where the deficiency of one component can be overcome through the use of another food product rich in such nutrient. Hence the development of composite fermented dairy products such as fermented milk with cereals can be a suitable option for improving the nutritional value and functionality of fermented milk products.

Finger millet is reported to be an economical millet with practically no reports of its adverse effects. It is rich in several micronutrients, phytonutrients and dietary fibre (*7*). Therefore, it can be a good ingredient for use with milk for developing a milk cereal functional food. Recently, considering the great potential of millet to contribute substantially to food and nutritional security of the country, the Indian Government declared finger millet along with other types of millet as “nutri-cereals” for production, consumption and trade (*8*). Finger millet is known for its health benefit properties such as antimicrobial, antioxidant, cholesterol lowering, blood glucose lowering effect, nephroprotective and anti-cataractogenic effect (*9*-*11*). High dietary fibre and phenolic content as well as low glycemic index make finger millet advantageous for diabetic patients (*12*, *13*). It is reported that consuming foods made of finger millet regularly can decrease fasting glucose by 32% and remove insulin resistance by 43% (*11*). Malting and fermentation of finger millet decrease its antinutritional factors, improve its carbohydrate digestibility and glycaemic response due to the transformation of starch to dextrins and maltose during germination (*11*). Phenolic extracts obtained from seed coats of finger millet were found to effectively inhibit the enzymes α-glucosidase and pancreatic amylase (*14*). Finger millet is rich in calcium (350 mg/100 g) and magnesium (137 mg/100 g). Researchers have reported a potential beneficial role of dietary calcium intake in reducing the risk of type 2 diabetes (*15*). At the same time a weaker association between magnesium intake and diabetes risk has been reported (*16*).

Many *in vitro* and *in vivo* study reports are available on the health benefits of finger millet and probiotic fermented milk, but these works mainly focused on antidiabetic potential of seed coat matter of finger millet and probiotic milk separately. We were unable to find any research work on antidiabetic potential of a food product prepared by fermentation of milk and finger millet using probiotic starter. This investigation evaluates the antidiabetic potential of probiotic fermented milk enriched with finger millet through *in vivo* animal study.

## MATERIALS AND METHODS

### Bacterial strains and their activation

Starter culture (comprising *Streptococcus thermophilus* MTCC 5460 and probiotic strain *Lactobacillus helveticus* MTCC 5463) was obtained from the culture collection of Dairy Microbiology Department, SMC College of Dairy Science, Anand Agricultural University, Anand, Gujarat, India. *S. thermophilus* MTCC 5460 is an isolate obtained from traditional curd sample and *L. helveticus* MTCC 5463 is a human vaginal isolate from Anand, Gujarat, India. Both cultures were propagated in sterilized reconstituted skimmed milk (10% total solids) medium by incubation at (37±1) °C for 6 h and stored at (5±2) ºC. The cultures were given three successive transfers in the same medium prior to their use to ensure the activity of cultures during the course of study.

### Preparation of test products

Test products used in the study include finger millet-enriched probiotic fermented milk (further in text: millet-enriched milk), probiotic fermented milk without finger millet (further in text: fermented milk), and malted finger millet flour (from now on: millet flour).

#### Preparation of malted finger millet flour

Malting of finger millet (variety PRM 9802, dark brown colour, obtained from a local market, Anand, Gujarat, India) was carried out as per the method optimized by Shaikh *et al.* (*17*). The cleaned grains were steeped in water for 12 h at (30±2) °C. The grain/water ratio was kept at 1:3. During steeping, water was changed every 4 h. Millet grains were hanged to remove excess water. Steeped grains were spread on perforated trays lined with muslin cloth and germinated at (25±2) °C for 24 h in an incubator. The grains were then uniformly spread on a stainless steel tray and vacuum dried in a tray dryer (Perfect Engineering and Allied Works Pvt. Ltd., Vadodara, Gujarat, India) maintained at 40-45 °C for 5 to 6 h. The malted, dried finger millet seeds were ground and made into flour using commercial flour mill (Milcent Super Flour Mill, Anand, Gujarat, India). Subsequently, the flour was sieved and stored in air-tight containers at (7±1) °C. The malted finger millet flour was heated at 70 °C and then added to the milk during product preparation.

#### Preparation of finger millet-enriched probiotic fermented milk

Milk (3.0% fat, 8.5% solids-not-fat) was heated to 90 °C for 5min, then cooled to 45 °C and 20% (*m*/*V*) heated malted finger millet flour was added. It was mixed to ensure uniform mass. The mixture of milk and millet was heated to 70 °C, then immediately cooled to 40 °C and inoculated with 2% culture (*Streptococcus thermophilus* MTCC 5460 and probiotic strain *Lactobacillus helveticus* MTCC 5463). Incubation was carried at (37±1) °C until a titratable acidity of about 0.7% lactic acid was obtained. The obtained product was then cooled to (5±1) °C and the curd was broken to obtain a uniform viscous product. It was filled in PET bottles and stored at (7±1) °C. Probiotic fermented milk prepared without finger millet and malted finger millet flour served as controls. The enriched milk and probiotic fermented milk were then freeze dried (Freeze Drying Systems Pvt. Ltd., Vadodara, Gujarat, India). The millet flour was used without pretreatment. Test formulations for feeding the rats were prepared freshly every day by suspending probiotic fermented milk samples with or without finger millet and malted finger millet flour in 0.5% carboxymethylcellulose (CMC) suspension.

### Sensory, microbiological, chemical and compositional evaluation of products

Titratable acidity, pH, probiotic and streptococcal count, sensory attributes and composition of the enriched milk and the probiotic fermented milk were analyzed using standard methods. Titratable acidity was determined after mixing 10 g of samples with 10 mL of distilled water and titrated against 0.1 M NaOH using 1% (*m*/*V*) phenolphthalein (BDH Prolabo, VWR Lab Products Private limited, Bangalore, India) as an indicator. The results were expressed as percentage of lactic acid (*18*). The pH of the products was measured using a pH meter (Oakton pH 700 benchtop meter; Vernon Hills, IL, USA).

For the culture count, 11 g of product were aseptically weighed and transferred to 99 mL sterile phosphate buffer (A.B. Enterprise, Mumbai, India) to obtain 1:10 dilution. Subsequently, 1 mL of the dilution was used for making further dilutions in 9-mL phosphate buffer tubes. Suitable dilutions were prepared and poured in a set of sterile Petri dishes in duplicates. For the enumeration of probiotic bacteria, 1.0 mL of selected dilutions were poured in duplicate plates and mixed with sterile cooled MRS agar (HiMedia Laboratories Pvt. Ltd, Mumbai, India). After setting of the agar, another layer of the same medium (5-7 mL) was poured. The plates were then incubated at (37±2) °C for 72 h. After incubation, the typical *Lactobacillus* colonies in the plates were counted and the count was expressed as log CFU/g (*19*). For the enumeration of streptococci, in place of MRS agar, M17 agar (HiMedia Laboratories Pvt. Ltd) was used.

Fermented products were subjected to sensory evaluation by expert panel of judges (*N*=8; four males (age range 25 to 60) and fourfemales (age range 25 to 50)) of various sensory attributes, *viz*. flavour, body and texture, acidity, colour and appearance, and overall acceptability criteria using 9-point hedonic scale described by Stone and Sidel (*20*). Coded samples of freshly prepared products were given to the panel of judges, who were asked to rank the products according to their preference using 9-point hedonic scale rating.

Fat in the product was determined by Rose-Gottlieb method (*21*). Protein content was determined by following macro-Kjeldahl method (*22*). Ash content of all the samples was determined by the procedure described in BIS handbook (*23*). Briefly, 20 g of homogenous sample were weighed in a clean and dry silica crucible. The sample in the crucible was heated on an open flame until it was completely reduced to ash. The sample was then transferred to a muffle furnace maintained at (550±2) °C. After cooling in the desiccator, the crucible was weighed. The process was repeated until constant mass. The total ash content in percentage was calculated as follows:


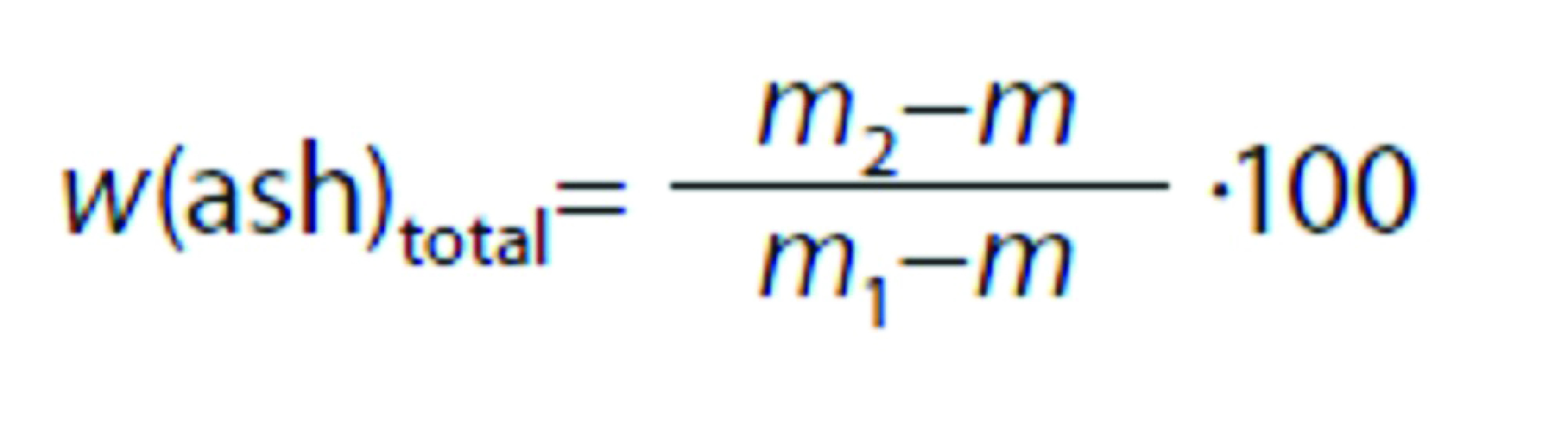


where *m* is the mass of the empty crucible (in g), *m*_1_ is the mass of the crucible with a sample (in g), and *m*_2_ is the mass of the crucible with ash (in g).

Total solid content of milk was estimated by gravimetric method (*24*). Calcium content of the samples was determined by the procedure described in BIS handbook (*23*). Briefly, 25 mL of ash solution were pipetted into a 250-mL beaker and diluted to about 50 mL with distilled water. Four to five drops of methyl red indicator were added and the pH was adjusted to 5.6 by the addition of freshly prepared ammonium hydroxide (S D Fine-Chem Limited, Mumbai, India). The beaker was heated on a sand bath and while it was still hot, 10-mL saturated ammonium oxalate (Merck Specialities Private Limited, Worli, Mumbai, India) solution was added drop by drop until the appearance of precipitate. The beaker was allowed to remain on the sand bath for 3 h or longer. The clear solution was decanted through Whatman No. 42 filter paper (Sigma-Aldrich, supplied by Merck Life Science Private Limited, Mumbai, India). The beaker in which the sample was originally precipitated as well as the precipitate on the filter paper were washed several times using hot distilled water to remove chloride and oxalate completely. The filter paper along with the precipitate was returned to the original beaker in which precipitation was done. A volume of 25 mL of 1:4 diluted sulfuric acid (Surya Fine Chem, Vadodara, Gujarat, India) was added to dissolve the precipitate. It was then heated to 60 °C, and while still hot, it was titrated against 0.01 M potassium permanganate (Madhu Chemicals, Vadodara, Gujarat, India) until the appearance of permanent pink colour. Calcium mass fraction was calculated using the following formula:


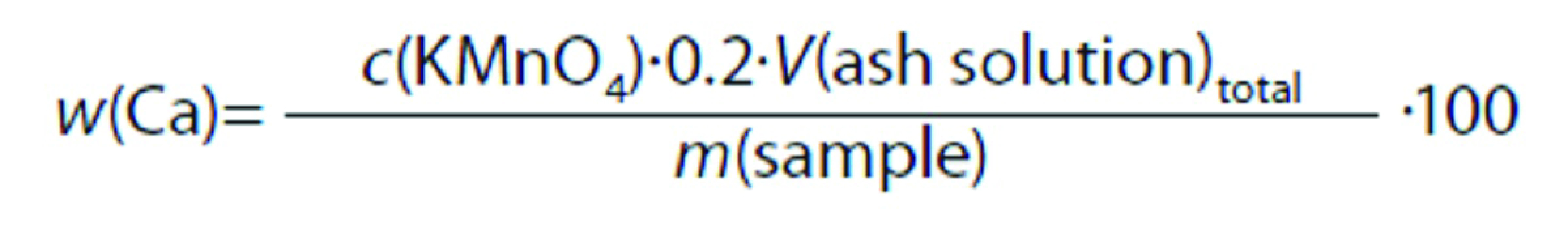


### Animal model and experimental design

The experimental design was approved by Institutional Animal Ethical Committee (IAEC) as per the guidance of Committee for the Purpose of Control and Supervision of Experiments on Animals (CPCSEA), Ministry of Social Justice and Empowerment, Government of India (Protocol No. APC/2017-IAEC/1721). Sprague Dawley rats (male) weighing 180-200 g were used in the study. The rats were housed in polypropylene cages and were kept under constant temperature ((20±5) °C) and humidity ((55±5) %) with a regular 12 h light/12 h dark cycle during the study. They were fed on standard pellet chow (VRK Nutritional Solutions, Pune, India) and were provided with water *ad libitum*. Standard pellet chow consisted of (in %): crude protein 18.15, crude fat 3.4, crude fibre 4.0, calcium 1.19, phosphorus 0.51, total ash 5.65, moisture 8.35 and carbohydrates 6.4.

For the induction of diabetes mellitus in experimental rats, the animals were fasted overnight and all the rats, except nondiabetic control and vehicle control (rats administered 0.5% CMC suspension) groups were administered a single intraperitoneal injection of freshly prepared solution of streptozotocin (STZ; Sigma-Aldrich, Merck, St. Louis, MO, USA) in 0.1 M citrate buffer (pH=4.5) at the dose of 55 mg/kg. Diabetes was assessed in rats by monitoring the fasting blood glucose (FBG) level 48 h after the injection of STZ using the glucose estimation kit (CPC Diagnostics, Chennai, India). Experimental rats with a blood glucose level of >13.88 mmol/L were considered as being diabetic and were used in the study.

Animals were randomized on the basis of their body mass and blood glucose level and divided into seven groups: (*i*) nondiabetic control, (*ii*) vehicle control, (*iii*) diabetic control (rats administered a single dose of 55 mg/kg STZ intraperitoneally), (*iv*) rats administered 55 mg/kg STZ and 45 mg/kg standard drug metformin, (*v*) freeze dried probiotic fermented milk (160 mg/500 g of rat), (*vi*) finger millet flour (40 mg/500 g of rat), and (*vii*) freeze dried probiotic fermented milk enriched with finger millet (200 mg/500 g of rat). Each group had 6 rats.

### Biochemical analysis

Food intake and water intake were recorded daily, while body mass of each group was recorded on weekly basis. At the end of 29 days, the animals were fasted overnight, and on the 30th day, blood was withdrawn by retro-orbital plexus method under the anesthetic (mixture of ketamine 75 mg/kg and xylazine 25 mg/kg; Merck, Mumbai, India) condition. Blood sample was collected in sterile Eppendorf tubes and centrifuged at 8000·*g* for 10 min using refrigerated centrifuge (Plastocraft, Mumbai, India) at 4 °C to get the serum and used for various biochemical studies. Biochemical parameters were determined by using auto-analyzer (Turbochem, Kolkatta, India). Animals were sacrificed using excessive dose of phenobarbitone, and liver and pancreas were preserved in 10% formalin for histopathological examination. Blood glucose, total cholesterol (T-CHL), high-density lipoprotein (HDL), low density-lipoprotein (LDL), triglycerides (TG), aspartate transaminase (AST) and alanine transaminase (ALT) levels were estimated by using standard enzymatic kits (CPC Diagnostics, Chennai, India). Very-low-density lipoprotein (VLDL) was calculated by using Freidewald's formula:

VLDL=TG/5 /3/

### Histopathological analysis

The tissue samples of liver and pancreatic sections from the experimental rats were fixed in 10% buffered formalin and embedded in paraffin. Tissues were sectioned (5 μm thickness) with microtome (Labindia instruments, Thane, India), fixed in slides and stained with haematoxylin and eosin (Everon Life Sciences, New Delhi, India). The slides were observed under trinocular microscope (Olympus, Ahmedabad, India) and photomicrographs were taken (10×).

### Statistical analyses

The results were expressed as mean value±standard error of the mean (SEM) and were analyzed statistically with one-way ANOVA followed by Dunnett’s *post-hoc* multiple comparison test using GraphPad Prism v. 6.00 (*25*). Values with p<0.05 were considered statistically significant.

## RESULTS AND DISCUSSION

Consumer interest in the functional foods that can combat non-communicable diseases like diabetes is rising. This research investigated the antidiabetic potential of a food product containing milk and millet. In the development of such foods, along with its specific health effects, the sensory attributes and probiotic count in the product are very important. In this study, throughout the experiment, the average viable count of the probiotic strain in probiotic fermented milk containing finger millet and probiotic fermented milk remained between 8.13 and 8.35 log CFU/g and the pH varied between 4.77±0.09 and 4.78±0.08; data not shown. Both products were sensorially acceptable (hedonic scale rating (8.43±0.16) for probiotic fermented milk containing finger millet and (7.64±0.24) for probiotic fermented milk; data not shown) as judged by an expert panel. Table 1 shows the average composition of the test products. The probiotic fermented milk with finger millet was sensorially acceptable, it contained the required level of probiotic count, and the incorporation of finger millet contributed to its dietary fibre, iron and calcium contents.

**Table 1:** Composition of the administered substances

Administered substance	*w*(total solid)/%	*w*(ash)/%	*w*(fat)/%	*w*(protein)/%	*w*(carbohydrate)/%	*w*(Ca)/(mg/100 g)	*w*(Fe)/(mg/100 g)	*w*(dietary fibre)/ (g/100g)
Millet-enriched milk	(24.6 ±0.1)^b^	(0.90 ±0.02)^b^	(3.07 ±0.05)^b^	(4.24 ±0.04)^b^	(16.3 ±0.2)^b^	(164.0 ±2.3)^b^	(2.20? ±0.12)^b^	(2.5)^b^
Fermented milk	(11.5 ±0.2)^c^	(0.61 ±0.02)^c^	(3.07 ±0.03)^b^	(3.04 ±0.02)^c^	(4.8 ±0.2)^c^	(114.1 ±2.5)^c^	N.D.	N.D.
Millet flour	(89.8 ±0.2)^a^	(2.9 ±1.2)^a^	(1.02 ±0.04)^a^	(7.70 ±0.02)^a^	(76.2 ±0.2)^a^	(384.0? ±0.2)^a^	(10.9 ±0.1)^a^	(12.8 ±0.1)^a^

N.D.=not determined. Values are mean±standard deviation of five replications. Mean values with different letters in superscript in the same column indicate significant differences (p<0.05)

### Induction of diabetes

Basal blood glucose level of all experimental rats on zero day varied between 6.57 and 6.97 mmol/L and the difference in the values between groups was found to be statistically insignificant (data not shown). The blood glucose level estimation on the third day after streptozotocin (STZ) administration for the confirmation of induction of diabetes in the experimental animal groups revealed a highly significant (p<0.0001) increase in the blood glucose level (21.26 to 22.81 mmol/L) of all experimental groups except nondiabetic control ((6.53±0.2) mmol/L) and vehicle control ((6.66±0.15) mmol/L) groups. The diabetogenic effects obtained due to STZ in this study are in accordance with the previous studies which showed that STZ in rats is associated with hyperglycaemia. STZ has a higher β-cell specificity relative to other diabetogens and selectively destroys the pancreatic β-cells that secrete insulin, which in turn decreases the level of insulin in the body and thus affects the normal sugar metabolism causing hyperglycaemia (*26*, *27*).

### Effect on body mass, food intake and water intake

**Fig. 1:**
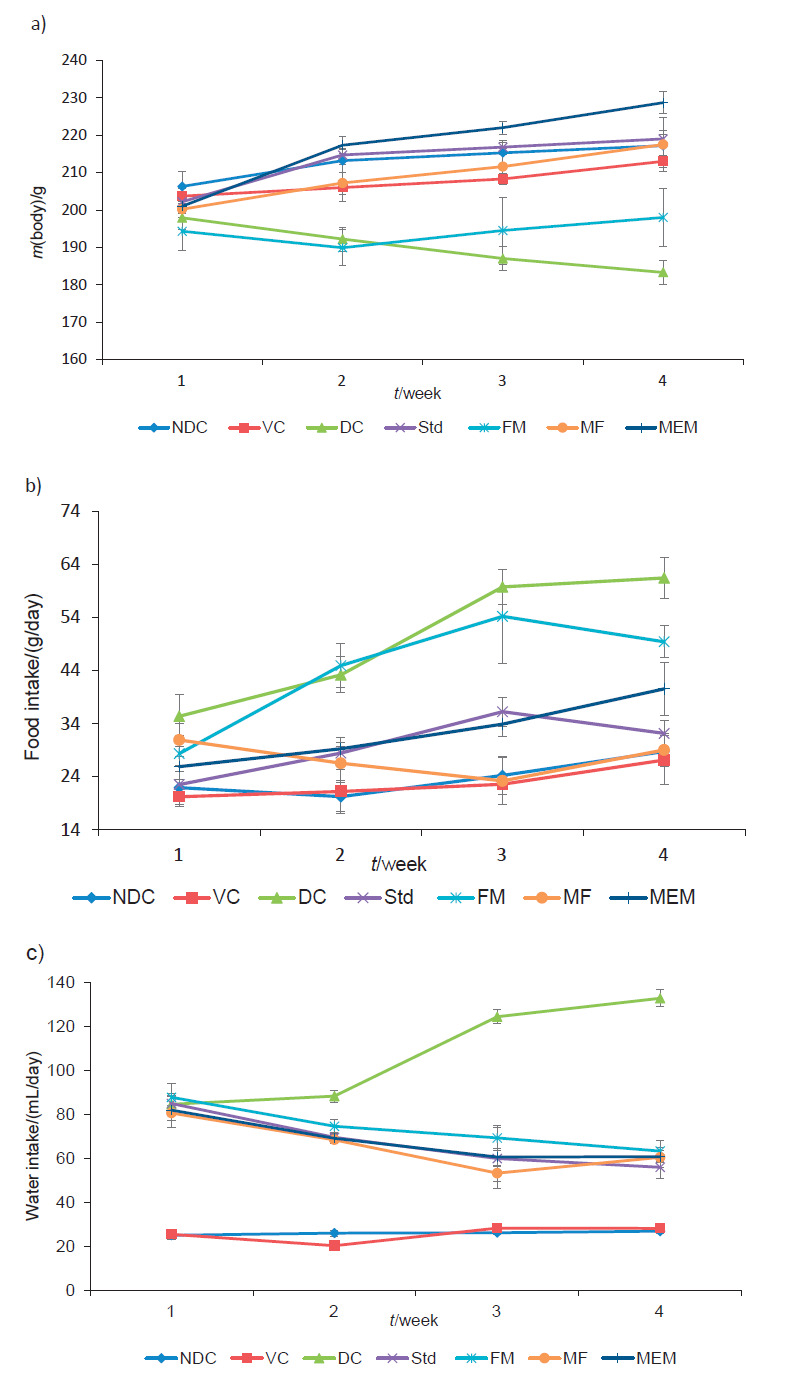
Effect of finger millet-enriched probiotic fermented milk on: a) body mass, b) food intake (g/day) and c) water intake (mL/day) of experimental rats. NDC=nondiabetic control, VC=vehicle control, DC=diabetic control, Std=diabetic rats administered metformin, FM=fermented milk, MF=millet flour, MEM=millet-enriched milk. Values are expressed as mean±SEM

Diabetes is usually associated with body mass loss due to impaired metabolic activities, irregular absorption and elimination of wastes and also because the body switches to burning fatty acids due to insulin shortage (*28*). The effects of four administered substances on body mass, food intake and water intake of the experimental rats are shown in Fig. 1a, Fig. 1b and Fig. 1c respectively. Diabetic control group showed significant (p<0.0001) decrease in body mass ((183.3±01.8) g), significant (p<0.01) increase in food intake ((61.5±3.9) g/day) and water intake ((142.7±3.8) mL/day) throughout the study compared to the nondiabetic control group (body mass (217.2±3.9) g, food intake (28.8±0.6) g/day, water intake (26.8±0.9) mL/day). An attenuation in the excessive food intake (polyphagia), excessive water intake (polydipsia) and body mass effects were seen in the groups with administered metformin, finger millet flour and millet-enriched probiotic milk as shown in Fig. 1b and Fig. 1c respectively. The millet-enriched probiotic milk and metformin groups showed significant (p<0.0001) increase in body mass ((228.1±2.9) and (219.0±1.2) g respectively)) compared to diabetic control rats. Nondiabetic control, vehicle control and groups administered finger millet flour also displayed an increase in body mass compared to diabetic control group, while the treatment with fermented milk caused insignificant decrease in the food intake ((49.5±3.0) g/day) and significant decrease in water intake ((113.2±4.8) mL/day) after 30 days. The millet-enriched probiotic milk was found to be more effective in alleviating these diabetic symptoms than the probiotic fermented milk and finger millet flour. Body mass in the diabetic control group was reduced up to 15.6% at the end of experimental protocol, whereas in the millet-enriched product group it increased by about 5-30%, which may be due to improved level of insulin in this group.

### Effect on fasting blood glucose level

**Fig. 2:**
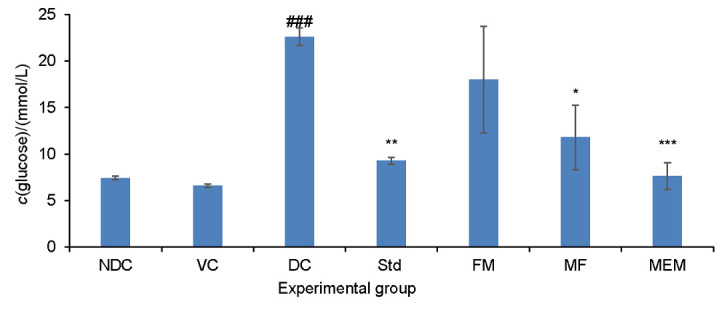
Effect of finger millet-enriched probiotic fermented milk on fasting blood glucose level of experimental rats. NDC=nondiabetic control, VC=vehicle control, DC=diabetic control, Std=diabetic rats administered metformin, FM=fermented milk, MF=millet flour, MEM=millet-enriched milk. Values are expressed as mean±SEM. #-Indicates significant difference from NDC group. *p<0.05, **p<0.01, ***p<0.001 indicate significant differences from DC group

Administration of the four substances orally for 30 consecutive days had a significant effect on serum glucose level (Fig. 2). Groups fed finger millet-enriched milk, metformin, and finger millet flour showed a significant decrease in blood glucose level. Finger millet-enriched milk group showed a highly significant (p<0.001) effect to the groups fed metformin (p<0.01) and finger millet flour (p<0.05), while fermented milk did not exert any significant decrease in fasting blood glucose level. The bioactive metabolites derived through the fermentation of milk-millet matrix may have contributed to the improved antidiabetic activity of finger millet-enriched milk compared to fermented milk and finger millet flour. Finger millet is reported to have hypoglycaemic effect owing to its high content of polyphenols, minerals and dietary fibre (*10*, *14*, *29*). Polyphenols such as caffeic acid, epigallocatechin-3-gallate and isoferulic acid are reported to enhance the uptake of glucose by peripheral tissues (*10*, *26*). Brown variety of finger millet is reported to contain more phenolic compounds compared to the white type (*10*). These phenolic compounds are found concentrated in the seed coat rather than in the flour fraction (*29*) and these compounds are reported to inhibit the intestinal α-glucosidase and pancreatic amylase to control postprandial hyperglycaemia. In our study, for the preparation of finger millet-enriched milk, we used flour prepared from brown variety of finger millet and the seed coat matter was retained in the flour. Diets enriched with finger millet seed coat matter were reported to be effective in ameliorating diabetic complications such as excretion of lower amounts of glucose, protein, urea and creatinine in addition to improving body mass in STZ-induced diabetic rats. Studies have reported that feeding a diet incorporated with 20% finger millet seed coat matter to STZ-induced diabetic rats caused 39% decrease in the fasting blood glucose value compared to diabetic control group (*26*, *27*). Another study reported that the germinated fermented finger millet had a higher level of total flavonoid, total phenol and free radical scavenging activity than the non-fermented one (*30*). Subastri *et al*. (*31*) in their study on koozh (a porridge made from germinated and fermented finger millet) reported an increased amino acid, phytochemical and free radical scavenging activity. In the case of a fermented milk with added millet, the bioactive peptides and amino acids from the milk proteins derived through fermentation might also have contributed to the positive role of finger millet polyphenols towards regulation of postprandial glycaemia and amelioration of complications related to diabetes. Studies have linked milk bioactive peptides with serum glucose regulatory effects in humans (*32*) and their proposed actions include an insulinotropic activity as well as the activity on different metabolic enzymes such as dipeptidyl peptidase IV, α-amylase and α-glucosidase, which are involved in the regulation of serum glucose (*33*). It is also reported that the free amino acids can directly affect the β-cell to release insulin (*34*).

The results of this study show that the hypoglycemic effect of probiotic fermented milk was not significant when compared to the millet-enriched probiotic milk, finger millet flour and metformin. Ruan *et al*. (*35*) in their meta-analysis of the effect of probiotics on glycaemic control reported a modest effect of probiotics on glycaemic control and pointed out that even a small reduction in fasting blood glucose (FBG) may provide significant public health benefits. It was also reported that the studies which used multispecies probiotic showed significant reduction in FBG in comparison to the studies that used single strain probiotic (*36*), and the probiotic effects are reported to be strain specific and influenced by the intricacy of the host microbiome interactions (*37*).

### Effect on lipid profile

**Fig. 3:**
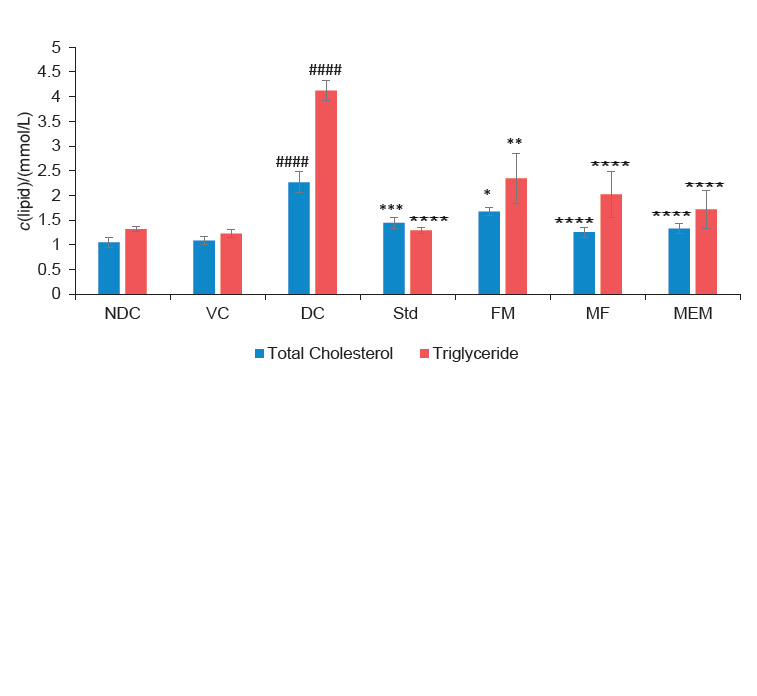
Effect of finger millet-enriched probiotic fermented milk on total cholesterol and triglyceride of experimental rats [NDC=nondiabetic control, VC=vehicle control, DC=diabetic control, Std=diabetic rats administered metformin, FM=fermented milk, MF=millet flour, MEM=millet-enriched milk. Values are expressed as mean±SEM; #-Indicates significant difference from NDC group; *p<0.05, **p<0.01, ***p<0.001, ****p<0.0001 indicate significant differences from DC group

**Fig. 4:**
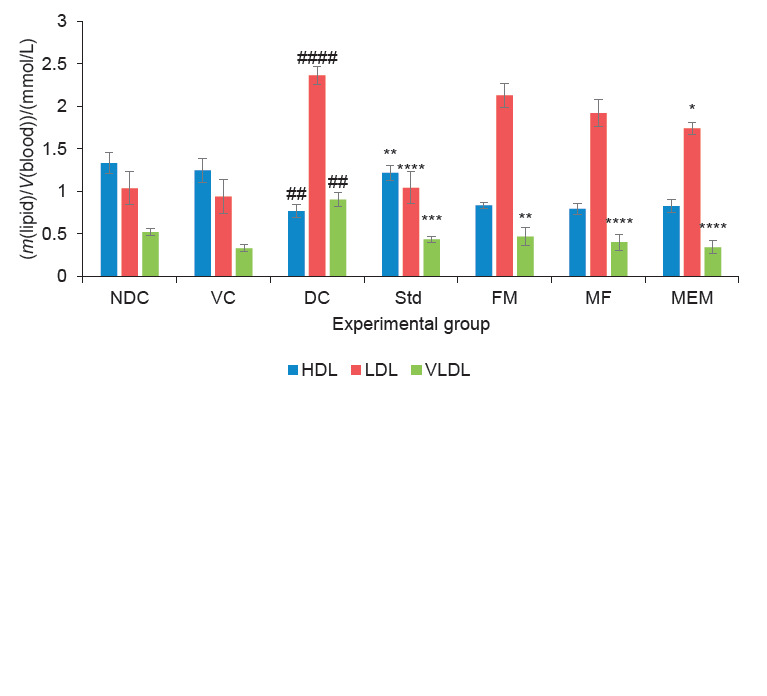
Effect of finger millet-enriched probiotic fermented milk on HDL, LDL and VLDL of experimental rats [NDC=nondiabetic control, VC=vehicle control, DC=diabetic control, Std=diabetic rats administered metformin, FM=fermented milk, MF=millet flour, MEM=millet-enriched milk. Values are expressed as mean±SEM. #-Indicates significant difference from NDC group. *p<0.05, **p<0.01, ***p<0.001, ****p<0.0001 indicate significant differences from DC group

Oral administration of test products caused hypolipidaemic action through significant reduction in T-CHL, TG, LDL and VLDL levels as shown in Fig. 3 and Fig. 4. A significant (p<0.001) increase in T-CHL and TG was seen in diabetic control group ((2.3±0.2) and (4.1±0.2) mmol/L respectively) compared to the nondiabetic control group ((1.1±0.1) and (1.3±0.1) mmol/L respectively), while a highly significant (p<0.0001) decrease in the levels of T-CHL and TG was observed in the groups administered the millet-enriched probiotic milk, finger millet flour and metformin. The probiotic fermented milk group also showed a significant (p<0.05) prevention in the rise of T-CHL and TG levels (Fig. 3). STZ injection caused a significant increase in LDL (p<0.001) and VLDL (p<0.01) levels but significant decrease in HDL (p<0.01) level, which was evident in diabetic control rats as compared to the nondiabetic control and vehicle control groups (Fig. 4). Oral administration of the millet-enriched probiotic milk, finger millet flour, probiotic fermented milk and metformin significantly decreased the VLDL level ((0.3±0.1), (0.4±0.1), (0.5±0.1) and (0.4±0.03) mmol/L respectively), but their levels of significance varied. As far as the LDL level is concerned, metformin exerted a highly significant (p<0.0001) decrease followed by the millet-enriched probiotic milk (p<0.05). The effect of probiotic fermented milk and finger millet flour was not found significant. Metformin-administered group showed a significant (p<0.01) increase in HDL level, which was not observed in the millet-enriched probiotic milk, probiotic fermented milk and finger millet flour groups. Overall, it can be seen that the millet-enriched probiotic milk significantly decreased the T-CHL, TG, LDL and VLDL levels (reduction of 41.44, 58.41, 26.32 and 62.07%, respectively) compared to finger millet flour (44.66, 50.98, 18.73 and 55.29%, respectively) and probiotic fermented milk (26.15, 43.21, 9.94 and 48.22%, respectively). Beneficial effect of the millet-enriched probiotic milk on blood lipid profile in comparison to controls might be due to the effect of probiotic fermentation of finger millet and milk. Our strain *L. helveticus* MTCC 5463 has been found to exert hypocholesterolemic effect in human subjects with different cholesterol levels (*38*). Research studies have shown that selected probiotic strains could inhibit dietary cholesterol absorption and suppress bile acid reabsorption in the small intestine (*39*). Such probiotic strains are found to produce short-chain fatty acids from indigestible carbohydrates of foods through fermentation. These short-chain fatty acids reportedly decreased cholesterol concentrations either through inhibition of hepatic cholesterol synthesis or by redistributing cholesterol from plasma to the liver (*40*). Selected studies have also pointed towards the protective effect of milk ingredients such as calcium and protein against hyperlipidaemia (*41*). Several studies have reported that probiotics could increase HDL-cholesterol (*42*, *43*). But in our study, we observed that the test products showed an insignificant rise in HDL level. Studies of Lee *et al*. (*44*) and Vasant *et al*. (*45*) highlighted the hypolipidaemic action of finger millet. The study by Lee *et al*. (*44*) reported that finger millet reduced total cholesterol and triglycerides in the serum of hyperlipidaemic rats. It was also reported that the soluble dietary fibre component of finger millet decreased reabsorption of bile acids (which are biosynthesized from cholesterol) and the LDL cholesterol (*46*, *47*).

### Effect on liver enzymes

**Fig. 5:**
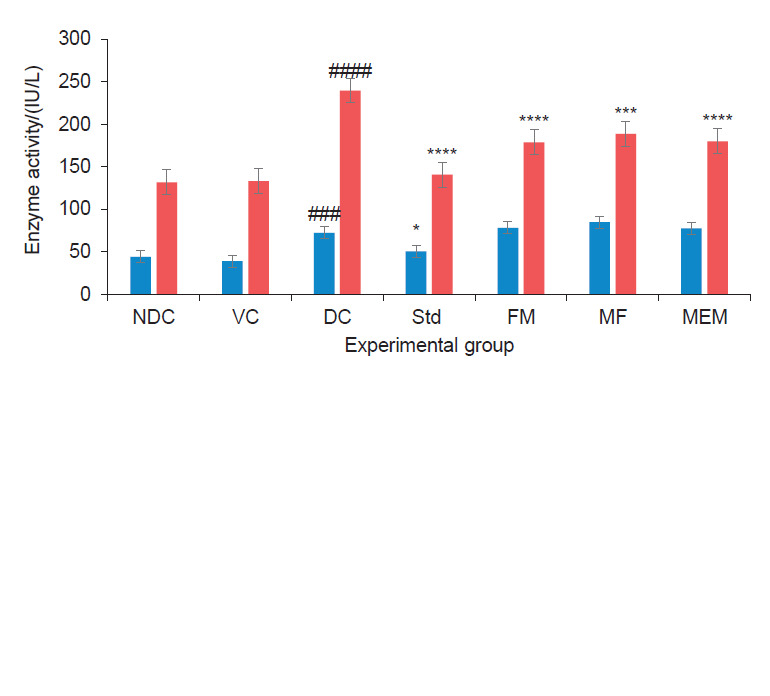
Effect of finger millet enriched probiotic fermented milk on liver enzymes aspartate aminotransferase (AST) and alanine aminotransferase (ALT) of experimental rats [NDC=nondiabetic control, VC=vehicle control, DC=diabetic control, diabetic rats administered metformin Std=diabetic rats administered metformin, FM=fermented milk, MF=millet flour, MEM=millet-enriched milk. Values are expressed as mean±standard error of mean. #-Indicates significant difference from NDC group. *p<0.05, ***p<0.001, ****p<0.0001 indicate significant differences from DC group

An increase of the activity of the two aminotransferases, alanine aminotransferase (ALT) and aspartate aminotransferase (AST), in the serum is one of the most regularly measured indicators of liver disease and occurs more often in diabetic patients than in the general population (*48*). These enzymes catalyze the transfer of amino groups to products in gluconeogenesis and amino acid metabolism and their elevated levels in the serum indicate acute or chronic liver injury (*49*). Our study results showed a significant (p<0.001) increase in the activity of ALT and AST ((239.6±5.8) IU/L) in diabetic control group compared to the nondiabetic control group ((131.5±0.5) IU/L). The millet-enriched product and probiotic fermented milk groups showed a highly significant (p<0.0001) decline in the activity of AST ((179.8±8.5) and (178.5±14.1) IU/L respectively), which was on a par with that of metformin ((140.6±5.2) IU/L). Finger millet flour group also exhibited a significant (p<0.001) decline in the AST activity. But except metformin, none of the treatments had shown a significant decline in ALT (Fig. 5). Significant decrease in liver enzyme activity could be ascribed to the polyphenolic content of the seed coat matter of finger millet as well as to the bioactive ingredients such as bioactive peptides in fermented milk. Okoyomoh *et al.* (*27*) assessed the antidiabetic properties of 20 and 40% seed coat matter of black finger millet in streptozotocin-induced diabetic male Wistar albino rats. They found that the different concentrations of seed coat matter had significantly protective effect by lowering the levels of ALT, AST and alkaline phosphatase (ALP) in serum.

### Effect on pancreas and liver histology

**Fig. 6:**
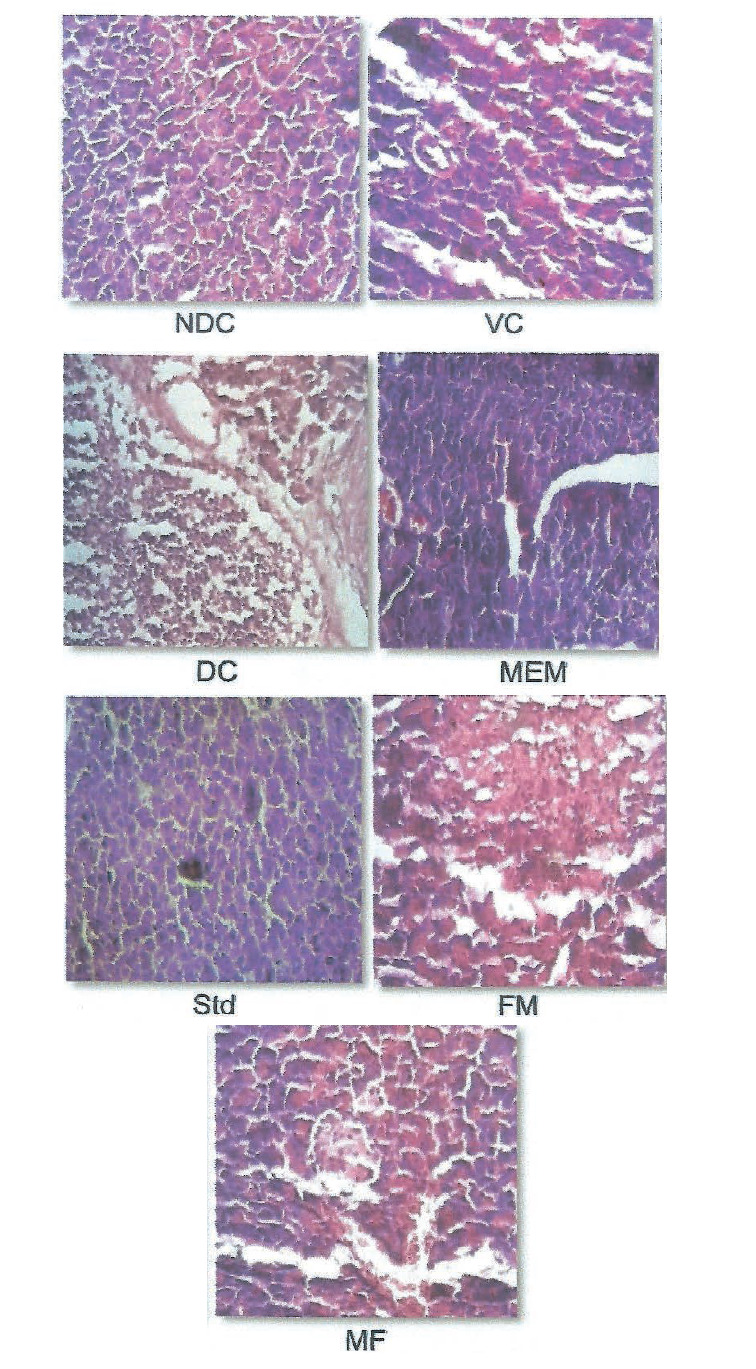
Light photomicrographs (hematoxylin and eosin staining, magnification: 10×) of pancreatic sections from different experimental groups. NDC=nondiabetic control, VC=vehicle control, DC=diabetic control, Std=diabetic rats administered metformin, FM=fermented milk, MF=millet flour, MEM=millet-enriched milk

**Fig. 7:**
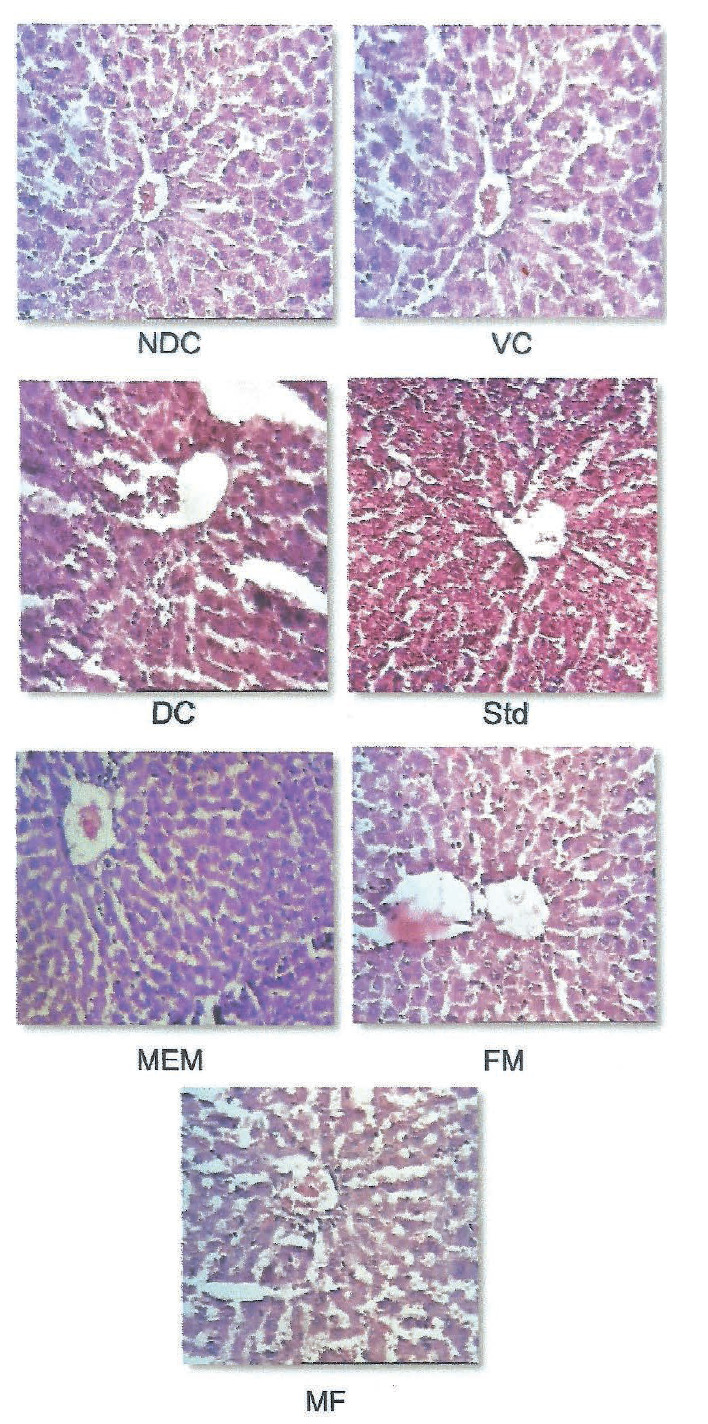
Light photomicrographs (Hematoxylin & Eosin, magnification: 10×) of liver sections from different experimental groups [NDC=nondiabetic control, VC=vehicle control, DC=diabetic control, Std=diabetic rats administered metformin, FM=fermented milk, MF=millet flour, MEM=millet-enriched milk

The effect of the four administered substances on rat pancreas and liver histology is shown in Fig. 6 and Fig. 7. Microscopic examination of pancreas section of the nondiabetic control and vehicle control rats showed normal architecture of the islets of Langerhans. The islets were stained lighter than the surrounding acinar cells. However, the diabetic control rats showed pathological changes in exocrine and endocrine components. The acinar cells were swollen and small vacuoles were observed in the majority of them. The β-cells of the islets are almost lost in STZ-treated rats. This is in agreement with studies demonstrating that a single dose of 55 mg/kg STZ is capable of inducing pancreatic β-cell destruction in rats and subsequent reduction of insulin secretion. On the other hand, administration of the four tested substances prevents more severe changes in the acinar cells. Moreover, destruction of β-cells is prevented, too. Targeting the pancreatic β-cells is considered one of the most promising strategies for treating diabetes (*50*). Microscopic findings of the liver of the nondiabetic control and vehicle control groups showed normal nuclei with sinusoidal cards around the central vein. STZ-diabetic rats revealed microcellular fatty changes, inflammation and mild vacuolization with disappearance of nuclei (Fig. 7), while administration of millet-enriched probiotic milk, finger millet flour and probiotic fermented milk ameliorated the inflammation and alteration in the liver structure to certain extent.

## CONCLUSIONS

Our study results have shown that finger millet (*Eleusine coracana*)-enriched probiotic fermented milk exerts remarkable antidiabetic and hypolipidaemic action in streptozotocin-induced diabetic rats. It was also effective in ameliorating other diabetic complications such as polyphagia and polydipsia and displayed a glucose-lowering action comparable to that of antidiabetic drug metformin. The enhanced antidiabetic potential of this composite food in comparison to probiotic and finger millet controls suggests that the fermentation of milk-finger millet matrix by probiotic culture might have a crucial role to play. The product can be a potential functional food in the prevention and management of diabetes, and it can be consumed as part of daily diet.
